# Comparison of different optomotor response readouts for visual testing in experimental autoimmune encephalomyelitis-optic neuritis

**DOI:** 10.1186/s12974-020-01889-z

**Published:** 2020-07-18

**Authors:** Christina Hecker, Michael Dietrich, Andrea Issberner, Hans-Peter Hartung, Philipp Albrecht

**Affiliations:** Department of Neurology, Medical Faculty, Heinrich-Heine University Düsseldorf, Moorenstr. 5, 40225 Düsseldorf, Germany

**Keywords:** Optomotor response, EAEON, Spatial frequency, Contrast sensitivity, Neurodegeneration

## Abstract

Optomotor response is increasingly used in preclinical research for evaluating the visual function in rodents. However, the most suitable measuring protocol for specific scientific questions is not always established. We aimed to determine the optimal parameters for visual function analysis in experimental autoimmune encephalomyelitis optic neuritis (EAEON), an animal model for multiple sclerosis. Contrast sensitivity as well as spatial frequency both had a low variance and a good test-retest reliability. Also, both parameters were able to differentiate between the EAEON and the control group. Correlations with the retinal degeneration, assessed by optical coherence tomography, the infiltration of immune cells, and the clinical disability score revealed that spatial frequency was superior to contrast sensitivity analysis. We therefore conclude that spatial frequency testing is better suited as visual acuity assessment in C57Bl/6 J EAEON mice. Furthermore, contrast sensitivity measurements are more time consuming, possibly leading to more stress for the animals.

## Introduction

Optic neuritis (ON) is a major source of disability in patients with inflammatory central nervous system diseases such as multiple sclerosis, and trials on optic neuritis are increasingly being applied to evaluate neuroprotective strategies [1]. Myelin oligodendrocyte glycoprotein-induced experimental autoimmune encephalomyelitis is reportedly associated with strong optic neuritis (EAEON) resulting in demyelination and axonal damage of the optic nerve and in the following degeneration of the inner retinal layers: retinal nerve fiber layer (axons), ganglion cell layer (neurons), and inner plexiform layer (dendritic arbor) [2–6]. Studies on preclinical models of experimental autoimmune encephalomyelitis optic neuritis (EAEON) are ideally suited to translationally evaluate the promise of clinical ON trials especially since more and more in vivo clinical readouts are being adapted to the preclinical setting [7]. To investigate the visual function in rodents, the optomotor response (OMR) can be used. The technique takes advantage of the optokinetic nystagmus, an involuntary tracking of a moving pattern. In rodents, the optokinetic nystagmus leads to reflexive movements of the head and neck, and therefore does not need any training of the animal. Nevertheless, mice need time to adapt to the experimental setting. In case of reduction or loss of visual function, the optomotor response is reduced or eliminated. Although the OMR is already used for visual function testing in rodents, the most sensitive and reliable OMR parameters are not well established. Spatial frequency (SF) and contrast sensitivity (CS) are often used as readouts to determine the thresholds of visual function.

In this study, we investigated if spatial frequency or contrast sensitivity analysis is better suited for the examination of visual function loss in myelin oligodendrocyte glycoprotein, fragment 35-55 (MOG_35-55_) induced EAEON in C57BL/6 J mice.

## Methods

### Induction of EAEON

Female, 6-weeks old C57Bl/6 J mice were purchased from Janvier Labs (Le Genest-Saint-Isle, France). To induce EAEON, the mice were subcutaneously immunized (distributed over four spots on the hind and front flanks) with 200 μg of MOG_35-55_ peptide (Biotrend, Cologne, Germany) in complete Freund’s adjuvant (CFA) (Becton Dickinson, Franklin Lakes, USA) containing 800 μg *Mycobacterium tuberculosis* H37Ra (Becton Dickinson, Franklin Lakes, USA). Additionally, the mice were intraperitoneally injected with 200 ng pertussis toxin (PT) (Sigma-Aldrich, St. Louis, USA) at the time of immunization and after 48 h. The sham control animals were immunized with phosphate-buffered saline in CFA and injected with the same PT doses. Clinical EAE scores were rated daily according to the following criteria: (0) no disease; (0.5) mild tail paresis; (1) obvious tail paresis or plegia; (1.5) tail plegia and no righting reflex; (2) mild signs of hind limb paresis with clumsy gait; (2.5) obvious signs of hind limb paresis; (3) hind limb plegia, mouse drags one leg behind; (3.5) hind limb plegia, mouse drags both legs behind; (4) mild signs of quadriparesis; (4.5) quadriplegia; and (5) death or moribund.

All performed animal procedures were done in compliance with the experimental guidelines approved by the regional authorities (State Agency for Nature, Environment and Consumer Protection; AZ 84-02.04.2016.A137)

### Analysis of visual function using optomotor response

Optomotor response was analyzed using a testing chamber and the OptoMotry™ software from CerebralMechanics™ (Canada) [8]. The mice were placed on a platform surrounded by four screens creating a box. The screens displayed a moving grid creating a virtual cylinder with varying frequencies at 100% contrast or varying contrasts at 5 different given frequencies (0.064, 0.092, 0.103, 0.192, and 0.272 c/d (cycles/degree)). The examination times differed substantially between contrast sensitivity and spatial frequency testing, the former taking about 60 min and the latter 15 min for a single exam. Monitoring was performed using a camera at the top of the box filming the head movements (tracking), which were evaluated by a blinded researcher. Visual acuity was determined using the threshold of the highest spatial frequency respectively the lowest contrast at which the mice still tracked the moving grid. Clockwise rotation of the grid and tracking represents the left eye while counterclockwise rotation and tracking represents the right eye. The baseline was analyzed before immunization and follow-up measurements were performed 1, 2, 3, 4, 6, 8, 10, 12, and 16 weeks post-immunization. A more detailed description of the device and methodology is given elsewhere [8–10].

### Optical coherence tomography

OCT measurements were performed under ambient light conditions using the Spectralis™ HRA + OCT device (Heidelberg Engineering, Heidelberg, Germany), and the methodology is reported in line with the APOSTEL recommendations [11]. The mice were anesthetized with isoflurane (Piramal critical care, Mumbai, India) vaporized at a concentration of 2.5% (2 L/min O_2_) and positioned in a custom OCT holder described elsewhere [12]. Pupils were dilated with 2.5% phenylephrine-0.5% tropicamide ophthalmic solution (pharmacy of the university hospital Düsseldorf). For imaging of the mouse retina, Visc-Ophtal eye gel (Dr. Winzer, Berlin, Germany) and a custom contact lens was used to keep the eyes moist and to ensure a constant and homogenous refraction during the examination. To adapt the focus to the mouse eye and retina, a 25-diopter adaptor lens was placed on the objective lens of the OCT device. OCT imaging was carried out with the software integrated TruTrack™ eye tracking to diminish breathing artifacts and to achieve consistent ocular orientations. OCT measurements were performed at the same time points as OMR analysis.

In order to analyze the thickness of the retinal layers, volume scans were performed. The scans were acquired with an initial focus of 37.75 diopters followed by manual correction. Each volume scan consisted of 25 B-Scans recorded in high-resolution mode at 50 automatic real time (ART, rasterized from 50 average A-Scans). The automated segmentation by the Heidelberg Eye Explorer™ software version 1.9.10.0 was followed by manual correction of obvious segmentation errors by a blinded investigator. Thickness measurements were derived from the circular 1, 2, and 3 mm early treatment of diabetic retinopathy study grid centered on the optic disc, excluding the central part. We calculated the thickness of the inner retinal layers (IRL), consisting of the retinal nerve fiber layer, ganglion cell layer, and inner plexiform layer by averaging each sector of the grid, excluding the center, which corresponded to the optic nerve head.

### Tissue sampling and histological analysis

One hundred ten days after immunization, mice were sacrificed with an overdose of isoflurane (Piramal critical care, Mumbai, India), and cardiac perfusion using phosphate-buffered saline (Gibco, Carlsbad, USA) was performed. Optic nerves were isolated and fixated with 4% paraformaldehyde (Carl Roth, Karlsruhe, Germany) overnight. Afterwards, the optic nerves were dehydrated in sucrose solutions with increasing concentrations and embedded in O.C.T. compound (Sakura™ Finetek, Alphen aan den Rijn, The Netherlands). Five micrometer longitudinal sections were cut, and hematoxylin and eosin staining was performed. Rating of infiltrating immune cells in optic nerves was performed by an investigator blinded to the experimental groups using a previously published score [13]: 0, no infiltration; 1, mild cellular infiltration; 2, moderate infiltration; 3, severe infiltration; and 4, massive infiltration.

### Statistics

Statistical analysis was performed using Prims 5.0 (Graphpad, San Diego, USA) and SPSS version 20 (IBM, Endicott, USA). Area under the curve of EAE daily scores was calculated for both groups and compared by *t* test analysis. Test-retest reliability was examined for spatial frequency and contrast sensitivity OMR measurements by interclass correlation coefficient (ICC) calculation using SPSS. Differences in retinal thickness and OMR measurements were analyzed using generalized estimating equations (GEE) with an exchangeable correlation matrix to adjust for intrasubject inter-eye correlations using SPSS. *P* values are selected as follows: *P ≤ 0.05, **P ≤ 0.01, and ***P ≤ 0.001.

## Results

### Test-retest reliability

**Fig. 1:**
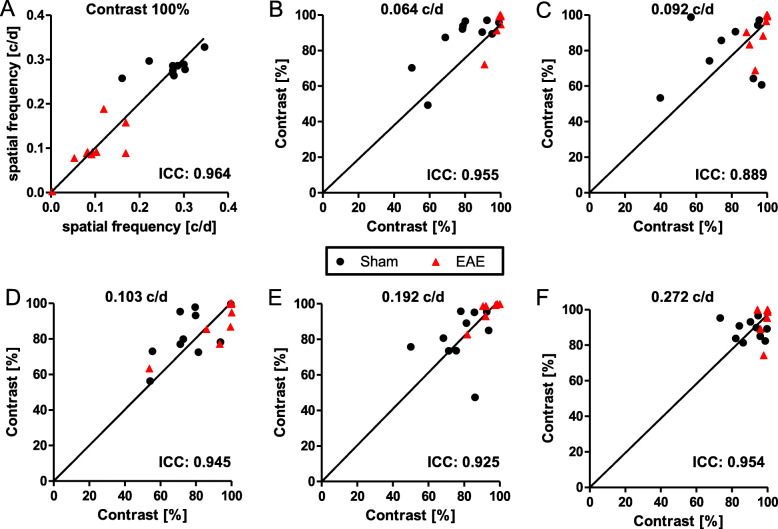
Retest analysis of visual acuity and contrast sensitivity show good reproducibility. Visual acuity analysis was performed at 100% contrast (**a**) and contrast sensitivity analysis at 0.064 (**b**), 0.092 (**c**), 0.103 (**d**), 0.192 (**e**) or 0.272 (**f**) cycles/degree (c/d). ICCs were calculated using SPSS (model: Two-way mixed; type: absolute agreement), (*n* = 10)

As a first step, the test-retest reliability of spatial frequency and contrast sensitivity measurements were investigated. Twenty eyes of 10 mice were tested on two consecutive days at the same day time by the same researcher 15 weeks post-immunization. Results showed that both measurements have a low variance and a good reproducibility with ICC values of 0.964 for spatial frequency (Fig. 1a) and 0.889–0.955 for contrast sensitivity (Fig. 1b–f).We were able to demonstrate that the best reproducibility to analyze contrast sensitivity is at a spatial frequency of 0.064 c/d. Although all readouts show good reproducibility, the animals present a considerable inter subject variability suggesting that longitudinal investigations analyzing changes from baseline are better suited to detect subtle changes than cross-sectional end point measurements. This is similar to the findings for OCT imaging and highlights the advantage of these in vivo assessments over other techniques like histology that are only possible post-mortem. The test-retest analysis revealed that spatial frequency testing is better suited to differentiate between control and EAE animals (Fig. 1a). Since in the context of EAEON the spatial frequency often drops below a value of 0.1 c/d (Fig. 1a), we decided to proceed with the contrast sensitivity analysis at 0.064 c/d for the following experiments.

**Fig. 2:**
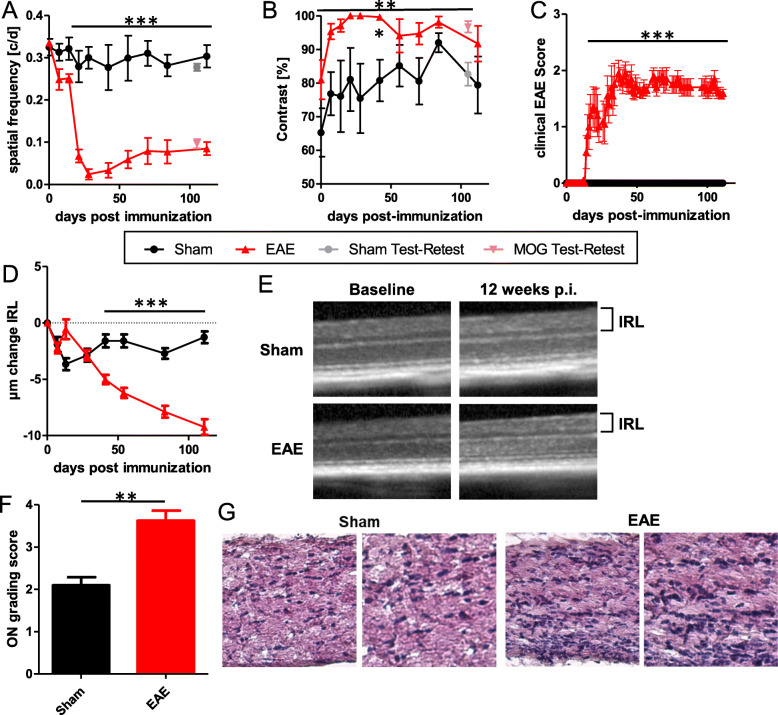
Analysis of visual function using OMR, thinning of the inner retinal layers and infiltration of immune cells in the inflammatory EAEON-model over a period of 110 days. Visual acuity tested by spatial frequency (**a**) and contrast sensitivity (**b**) at 0.064 c/d (n = 4, note that a higher contrast equals worse visual acuity; compared by GEE analysis). **c** Clinical EAE score (area under the curve compared by t-test), **d** change of inner retinal layers (compared using GEE analysis), **e** representative OCT images (p.i. post-immunization), **f** infiltrating immune cells in the optic nerve (*n* = 5), and **g** representative histological images of optic nerves (H&E staining). The time courses present the results of an EAE experiment with at least four mice per group (*p < 0.05; **p < 0.01; ***p < 0.001)

We continued by testing both readouts in the EAEON mouse model with eight eyes of two sham/control and two EAEON mice. In general, spatial frequency and contrast sensitivity analysis were able to differentiate between sham/control and EAEON mice. However, spatial frequency showed not only a clearer separation of the two groups (Figs. 1a and 2a + b), but also a better correlation with the degeneration of the IRL, assessed by OCT (Fig. 2d + e) (SF, *p* < 0.001 vs CS, *p* < 0.001; GEE), infiltrating immune cells (Fig. 2f + g) (SF, *r* = − 0.78; *p* = 0.028 vs CS, *r* = 0.42; *p* = 0.354; Spearman), and the EAE score (Figure 2c) (SF, *r* = − 0.76; *p* < 0.001 vs CS, *r* = 0.38; *p* = 0.059; Spearman) than contrast sensitivity.

## Discussion

In this study, spatial frequency and contrast sensitivity analysis were compared as readout methods to examine visual acuity in EAEON mice. Furthermore, correlation with clinical EAE-scores, thinning of inner retinal layers, and infiltrating immune cells in the optic nerve was studied. Even though OMR is a reflex and therefore training is not mandatory, mice still need time to adapt to the experimental setting. During long-time measurements, such as measurements of all five frequencies for contrast sensitivity analysis, animals sometimes need to be animated to differentiate if loss of tracking is due to reaching the threshold or due to decreasing attention of the animal. Furthermore, experimenters should be blinded for the experimental groups and need training to reliably distinguish between tracking and normal behavioral movements. Choosing the right spatial frequency for contrast sensitivity testing is crucial. Visual function is continuously being lost during EAEON, and in order to assure reliable results over the whole course of the disease, we decided to perform the test-retest analysis with mice that were in a chronic stage. This may account for the worse separation of EAE, and sham-immunized mice in the contrast measurements at the higher spatial frequencies testing spatial frequencies between 0.064 c/d and 0.272 c/d for test-retest reproducibility and during EAEON revealed that 0.064 c/d was the most reproducible and was still detectable by mice even at lower contrast during EAEON. Testing at lower spatial frequencies was not possible for contrast sensitivity assessments due to the specifications of the device. Although other groups found that in rhodopsin knockout mice contrast sensitivity analysis was more sensitive for visual decline than spatial frequency [14], we conclude that for EAEON mice the opposite is true. These different conclusions highlight the fact that different models and reasons for visual deficits may need different algorithms for testing visual function. While rhodopsin knockout mice show decrease of cone density and retinal thinning of the outer retinal layers, our EAEON mice present neuroaxonal degeneration and thinning of the inner retinal layers and the optic nerve. It is therefore important to evaluate the sensitivity of different testing algorithms before starting visual testing in new experimental models. To this end, our data suggest that spatial frequency testing at 100% contrast and contrast sensitivity testing at 0.064 c/d seem to be good starting points, in line with results from other researchers [8, 15].

## Conclusions

We demonstrate that, in EAEON mice, spatial frequency measurement is better suited for the analysis of the optokinetic response than contrast sensitivity testing. Apart from the superior results for spatial frequency, analysis of contrast sensitivity is also more time-consuming and therefore leads to more stress for the mice and a lower throughput for experiments.

## Data Availability

The dataset obtained and analyzed in this study is available from the corresponding author on a reasonable request.

## Abbreviations

CFA: Complete Freund’s adjuvant
CS: Contrast sensitivity
EAEON: Experimental autoimmune encephalomyelitis/optic neuritis
GEE: Generalized estimated equation
ICC: Intraclass correlation coefficient
IRL: Inner retinal layers
MOG_35-55_: Myelin oligodendrocyte glycoprotein fragment 35-55
OCT: Optical coherence tomography
OMR: Optomotor response
ON: Optic neuritis
p.i.: Post-immunization
PT: Pertussis toxin
SF: Spatial frequency
